# High-density lipoprotein cholesterol levels is negatively associated with intertrochanter bone mineral density in adults aged 50 years and older

**DOI:** 10.3389/fendo.2023.1109427

**Published:** 2023-03-24

**Authors:** Pei Yang, DongDong Li, Xiaokang Li, Zongbiao Tan, Huan Wang, Xiaona Niu, Yang Han, Cheng Lian

**Affiliations:** ^1^ Department of Cardiology, Xi’an International Medical Center Hospital, Xi’an, Shaanxi, China; ^2^ Department of Cardiology, Tangdu Hospital, Air Force Medical University, Xi’an, Shaanxi, China; ^3^ Department of Gastroenterology, Renmin Hospital of Wuhan University, Wuhan, Hubei, China; ^4^ Department of Orthopaedic, Tangdu Hospital, Air Force Medical University, Xi’an, Shaanxi, China

**Keywords:** High-density lipoprotein cholesterol, bone mineral density, correlation, NHANES, race

## Abstract

**Background:**

High-density lipoprotein cholesterol (HDL-C) has long been viewed as a protective factor for cardiovascular health. Yet, higher HDL-C was not necessarily beneficial. The purpose of this study was to investigate the relationship between HDL-C levels and intertrochanter bone mineral density.

**Methods:**

The study collected the most recent data from the 2017-2020 National Health and Nutrition Examination Survey (NHANES). Weighted multiple regression analysis was used to evaluate the relationship between HDL-C and intertrochanter BMD, and further subgroup analysis and threshold effect analysis were conducted. Finally, the relationship between HDL-C and intertrochanter BMD was analyzed by fitting smooth curves.

**Results:**

The study included 3,345 people ranging in age from 50 to 80. HDL-C was discovered to be negatively correlated with intertrochanter BMD (β = -0.03, 95%CI: -0.04, -0.01, *P* = 0.0002). In subgroup analysis, the negative correlation was found among 60-70-year-olds (β = -0.04, 95%CI: -0.06, -0.02, *P* = 0.0010), additionally, non-Hispanic whites (β = -0.03, 95%CI: -0.05, -0.01, *P* = 0.0140), and obese individuals (β = -0.03, 95%CI: -0.05, -0.01, *P* = 0.0146). The negative correlation, on the other hand, remained significant and consistent across genders, menstruation status, hormone usage, and long-term use of steroids. The relationship between HDL-C and intertrochanter BMD was an inverted U-shaped curve in men and hormone users, with inflection points of 1.01 mmol/L and 1.71 mmol/L, and an U-shaped curve in other Hispanic and premenopausal individuals, with inflection points of 0.96 mmol/L and 1.89 mmol/L.

**Conclusions:**

HDL-C was negatively associated with intertrochanter BMD in people over 50 years of age, non-Hispanic whites, and obesity.

## Introduction

High-density lipoprotein cholesterol (HDL-C), which is mostly formed in the liver, is an anti-atherosclerosis lipoprotein that transfers cholesterol from extrahepatic tissue to the liver for processing and prevents atherosclerosis *via* antioxidant, anti-inflammatory, and other mechanisms (1). As a result, it has been the subject of widespread attention among doctors and older patients in the field of cardiovascular disease. In clinical practice, we observed that such elderly were indeed prone to osteopenia and osteoporosis (2), raising the question of whether so-called higher and better HDL-C protects against cardiovascular disease but also increased the likelihood of fractures in the elderly (3).

According to recent research, higher HDL-C levels are not necessarily healthier and are even connected with an increased risk of mortality in the general population (4–6). Some discoveed a favorable link between the two (7–11), some considered a negative correlation (12–14), while a few consider no relationship at all (15). Tang et al. identified a negative association between HDL-C and BMD that was significant in people aged 30-50 and 50-59 when segregated by gender but not in males (16). Xie et al. provided some useful relations among HDL-C and lumbar BMD, particularly notable in women and black men, and established a threshold value (0.98 mmol/L) in males and white men (7). Cui et al.’s work demonstrated that hdl-c does not have an association with lumbar BMD, which is not affected by the menstrual period (15). This ambiguous interaction was also variable in terms of demographic differences (gender, race, menopause, HRT, etc.) and various skeletal locations of bone density (lumbar, femoral neck, hip, etc.) (17, 18).

Therefore, we investigated the relationship between HDL-C levels and intertrochanter BMD in U.S. adults 50 years of age and older using the 2017-2020 National Health and Nutrition Examination Survey (NHANES) database and identified specific populations through stratified analysis and threshold effect analysis to provide a reference for clinical practice.

## Materials and methods

### Study population

**Figure 1:**
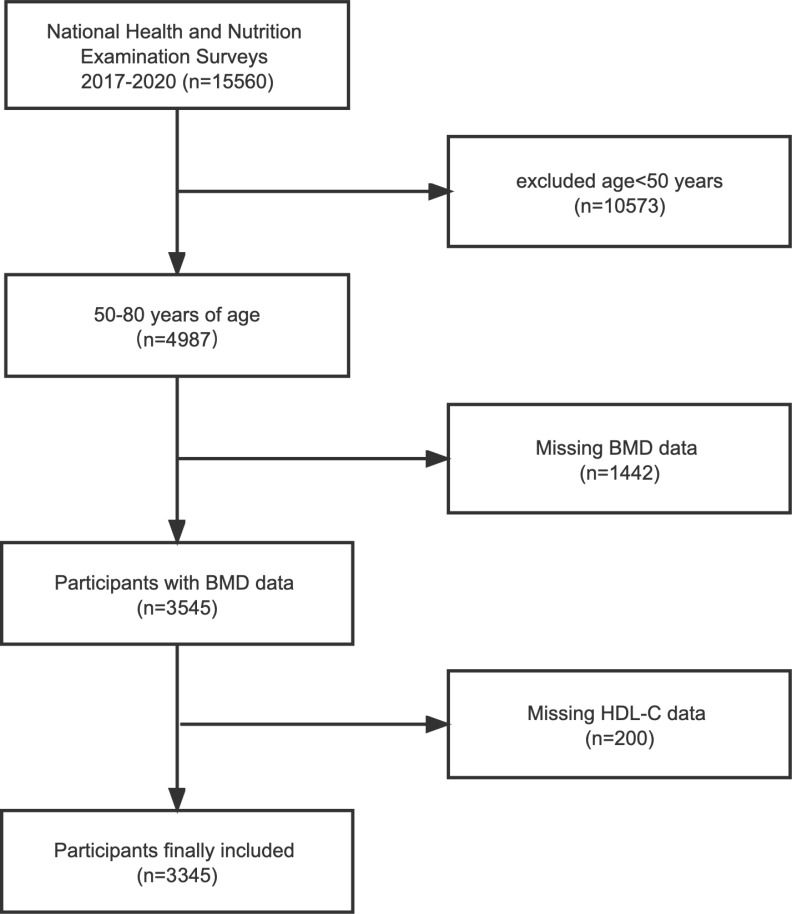
Flowchart of the sample selection from the 2017-2020 National Health and Nutrition Examination Survey (NHANES). BMD, bone mineral density; HDL-C, high-density lipoprotein cholesterol.

NHANES is a major, ongoing national cross-sectional study sponsored by the Centers for Disease Control and Prevention. Objective statistical data on health issues were obtained to assess the health and nutritional status of the general population of the United States using a complex multistage stratified sampling design. To explore the possible relationship between HDL-C and BMD, this investigation used NHANES data from the 2017-2020 cycle. Of the 15,560 participants, we excluded 10,573 patients younger than 50 years of age, 1442 with missing BMD data, and 200 with missing HDL-C data. In the end, a total of 3,345 people participated in the study (**Figure 1**).

NHANES was authorized by the NCHS Ethics Review Committee to perform any research procedures involving human individuals with signed informed permission from all participants. NHANES’ data was anonymized and made available for public use. We agree to follow the guidelines for data use in the study to ensure that the data are only used for statistical analysis and that all experiments meet applicable standards and regulations.

### Study variables

Questionnaires were collected at baseline to obtain demographic information (age, gender, race, marital status, and education), smoking status, exercise status, personal medical history (diabetes, tumors), etc. Body mass index (BMI) was calculated by dividing weight (kg) by the square of height (m^2^). Dual-energy X-ray bone mineral density was measured by the Hologic QDR 4500A bone mineral density analyzer and APEX 3.2 software. Blood samples were collected on an empty stomach to determine blood calcium, total protein, blood urea nitrogen, blood phosphorus, total cholesterol, high-density lipoprotein cholesterol (HDL-C), etc. Covariables in multivariate models may lead to confusion in the correlation between HDL-C and intertrochanter BMD. Age, gender, race, body mass index, marital status, exercise, smoking, diabetes, cancer, menstruation status, female hormone, long-term steroid usage, anti-osteoporosis treatment history, blood calcium, serum phosphorus, total cholesterol, and total protein were all covariates of this study. Detailed information on these covariates is publicly available on the website of the Ministry of Health and Social Welfare (www.cdc.gov/nchs/NHANES/).

### Statistical analysis

All statistical analyses were performed using R software (version 4.1.2), and weights were calculated as recommended by the analysis guide compiled by NCHS (19). For normally distributed variables, continuous variables are expressed as mean ± standard deviation; categorical variables are expressed as numbers (N) and percentages (%). All the null data of the covariables were replaced by the mean values, and the total number of missing values was less than 1%. The *P* values of the continuous variables in the population baseline table were calculated by the weighted linear regression model, and the *P* values of the classified variables were calculated by the weighted Chi-square test. The Pearson and Spearman correlation analysis was used to examine the relationship between covariates and BMD. A weighted multiple regression model was used to calculate the relationship between exposure factors and BMD, and no adjustment variables were found in model 1. Model 2 was adjusted for age, gender, and race. Model 3 was adjusted for age, gender, race, body mass index, marital status, exercise, smoking, diabetes, cancer, menstruation status, female hormone, long-term steroid usage, anti-osteoporosis treatment history, blood calcium, serum phosphorus, total cholesterol, and total protein. In addition, HDL-C was further converted from a continuous variable to tertiles for sensitivity analysis and trend testing. We used stratified multiple regression analysis for subgroup analysis by age, gender, race, BMI, menstruation status, female hormone, long-term steroid usage, and anti-osteoporosis treatment history. *P* value < 0.05 was considered statistically significant.

## Results

### Baseline characteristics

The characteristics of the population and laboratory results following the weighted inclusion of individuals are provided in **Table 1**. A total of 3345 participants were included, with an average age of 63.57 ± 8.82 years, of which 49.31% were male and 50.69% were female. The overall average value of HDL-C was 1.43 ± 0.43 mmol/L, separated into T1 (≤ 1.0), T2 (1.0-2.0), and T3 (> 2.0) acccording to the clinical range, and the mean values were 0.88 ± 0.10 mmol/L, 1.41 ± 0.26 mmol/L, and 2.32 ± 0.30 mmol/L, respectively, *P* < 0.0001. The total mean value of intertrochanter BMD was 1.10 ± 0.19 g/cm^2^, and the mean values were 1.19 ± 0.17 g/cm^2^, 1.10 ± 0.19 g/cm^2^, and 0.96 ± 0.17 g/cm^2^, respectively, *P* < 0.0001. The higher the HDL-C level, the lower the intertrochanter BMD. Among those with high HDL-C levels, the proportion of women was substantially higher than that of men. Those with low HDL-C levels were more likely to be smokers. Among women, 91.59% went through menopause after the age of 50, and 29.65% used female hormone therapy. 7.5% used steroids long-term, and 11.13% received anti-osteoporosis therapy.

**Table 1:** Weighted baseline characteristics of participants (N=3345).

Characteristics	HDL-C (mmol/L)				*P* value
	Total	T1(≤1.0)	T2(1.0-2.0)	T3(>2.0)	
No. of participants	n=3345	n=430	n=2602	n=313	
Age (years)	63.57 ± 8.82	62.67 ± 8.22	63.49 ± 8.89	65.39 ± 8.79	<0.0001
Gender (%)					<0.0001
Male	49.31	78.33	48.90	14.79	
Female	50.69	21.67	51.10	85.21	
Race and ethnicity (%)					0.0011
Mexican American	4.77	6.14	4.91	1.87	
Other Hispanic	6.61	7.27	6.86	3.85	
Non-Hispanic white	71.02	73.24	70.03	75.82	
Non-Hispanic black	9.03	5.37	9.26	11.98	
Other race/ethnicity	8.57	7.97	8.94	6.47	
Education level (%)					<0.0001
Less than high school	10.76	15.74	10.52	6.16	
High school	28.85	35.16	28.66	22.07	
More than high school	60.39	49.10	60.82	71.78	
Marital status (%)					<0.0001
Married/living with partner	65.86	75.02	64.83	61.98	
Widowed/divorced/seperated	28.31	16.42	29.58	33.86	
Others	5.83	8.56	5.59	4.15	
BMI (kg/m^2^)	29.30± 5.89	31.73 ± 5.84	29.34 ± 5.81	25.85 ± 4.77	<0.0001
Waist cricumference (cm)	102.10 ± 14.36	109.77 ± 13.80	102.12 ± 13.92	91.95 ± 11.97	<0.0001
Moderate activities (%)					0.0004
Yes	47.06	39.84	47.36	54.11	
No	52.94	60.16	52.64	45.89	
Smoked at least 100 cigarettes in life (%)					0.0017
Yes	45.33	53.30	44.07	44.73	
No	54.67	46.70	55.93	55.27	
Diabetes status (%)					<0.0001
Yes	18.44	40.90	16.37	5.39	
No	78.21	53.29	80.39	93.61	
Others	3.35	5.82	3.24	1.00	
Cancer or malignancy (%)					0.0095
Yes	19.74	24.71	19.35	16.39	
No	80.26	75.29	80.65	83.61	
Total Calcium (mmol/L)	2.33 ± 0.09	2.33 ± 0.10	2.33 ± 0.09	2.35 ± 0.08	<0.0001
Total Protein (g/dL)	7.03 ± 0.44	7.06 ± 0.43	7.03 ± 0.44	6.99 ± 0.45	0.0704
Blood Urea Nitrogen (mmol/L)	5.99 ± 2.04	6.30 ± 2.14	5.96 ± 2.00	5.86 ± 2.14	0.0030
Phosphorus (mg/dL)	3.57 ± 0.51	3.53 ± 0.52	3.56 ± 0.51	3.71 ± 0.49	<0.0001
Total cholesterol (mg/dL)	193.00 ± 42.92	169.77 ± 44.64	194.14 ± 41.43	214.25 ± 38.22	<0.0001
HDL-C (mmol/L)	1.43 ± 0.43	0.88 ± 0.10	1.41 ± 0.26	2.32 ± 0.30	<0.0001
Intertrochanter BMD (g/cm^2^)	1.10 ± 0.19	1.19 ± 0.17	1.10 ± 0.19	0.96 ± 0.17	<0.0001
Menstruation status (%)					
Premenopausal	5.63	2.23	6.34	3.45	0.1582
Postmenopausal	91.59	96.78	90.78	93.63	
Unknown	2.78	0.99	2.88	2.91	
Female hormone usage (%)					0.6383
Yes	29.65	29.25	28.94	33.05	
No	67.25	69.22	67.84	63.84	
Unknown	3.11	1.54	3.22	3.11	
Long-term steroid usage (%)					0.3736
Yes	7.50	5.53	8.06	5.58	
No	91.96	93.95	91.29	94.42	
Unknown	0.54	0.51	0.66	–	
Anti-osteoporosis treatment history (%)					0.1201
Yes	11.13	10.09	10.21	15.79	
No	9.62	10.60	9.87	8.11	
Unknown	79.25	79.32	79.92	76.10	

Mean ± SD for continuous variables: P value was calculated by a weighted linear regression model.

% for categorical variables: P value was calculated by weighted chi-square test.

The proportion of menstruation status, female hormone usage were only calculated in the female group.

HDL-C, high-density lipoprotein cholesterol; BMI, body mass index; BMD, bone mineral density.

Age, gender, race, education, marital status, BMI, waist circumference, exercise status, smoking status, diabetes status, tumor status, blood calcium, blood uric acid, blood phosphorus, and total cholesterol all differed significantly (all *P* < 0.05), but menstruation status, female hormone usage, long-term steroid usage, anti-osteoporosis treatment history, and total protein had no substantial change (*P* > 0.05). The results of covariates associated with Intertrochanter BMD are shown in **Table 2**.

**Table 2:** Univariate analysis for Intertrochanter BMD.

Outcome	Statistics	β (95%CI)	*P* value
Age (years)	64.42 ± 9.05	-0.01 (-0.01, -0.00)	<0.0001
Gender (%)			
Male	52.94	Reference	
Female	47.06	-0.17 (-0.18, -0.16)	<0.0001
Race and ethnicity (%)			
Mexican American	9.69	Reference	
Other Hispanic	11.27	-0.03 (-0.07, 0.01)	0.1109
Non-Hispanic white	39.22	-0.06 (-0.09, -0.03)	<0.0001
Non-Hispanic black	24.60	0.01 (-0.03, 0.05)	0.6070
Other race/ethnicity	15.22	-0.05 (-0.09, -0.02)	0.0048
Education level (%)			
Less than high school	19.52	Reference	
High school	25.08	-0.00 (-0.03, 0.02)	0.8243
More than high school	55.40	0.00 (-0.02, 0.02)	0.8179
Marital status (%)			
Married/living with partner	60.06	Reference	
Widowed/divorced/seperated	31.75	-0.07 (-0.09, -0.06)	<0.0001
Others	8.19	-0.00 (-0.03, 0.02)	0.7871
BMI (kg/m^2^)	29.18 ± 6.05	0.01 (0.01, 0.01)	<0.0001
Waist cricumference (cm)	101.61 ± 14.37	0.01 (0.00, 0.01)	<0.0001
Moderate activities (%)			
Yes	38.86	Reference	
No	61.14	-0.02 (-0.03, -0.01)	0.0052
Smoked at least 100 cigarettes in life (%)			
Yes	46.16	Reference	
No	53.84	-0.02 (-0.04, -0.01)	0.0005
Diabetes status (%)			
Yes	22.60	Reference	
No	73.42	-0.09 (-0.11, -0.07)	<0.0001
Others	3.98	0.03 (-0.01, 0.07)	0.1321
Cancer or malignancy (%)			
Yes	16.80	Reference	
No	83.20	0.06 (0.04, 0.07)	<0.0001
Total Calcium (mg/dL)	2.33 ± 0.10	-0.17 (-0.23, -0.10)	<0.0001
Total Protein (g/dL)	7.12 ± 0.45	0.04 (0.03, 0.06)	<0.0001
Blood Urea Nitrogen (mmol/L)	5.92 ± 2.21	0.00 (-0.00, 0.00)	0.5872
Phosphorus (mg/dL)	3.55 ± 0.53	-0.05 (-0.06, -0.04)	<0.0001
Total cholesterol (mg/dL)	190.01 ± 43.46	-0.00 (-0.00, -0.00)	<0.0001
HDL-C (mmol/L)	1.42 ± 0.42	-0.15 (-0.17, -0.14)	<0.0001
Menstruation status (%)			
Premenopausal	5.63	Reference	
Postmenopausal	91.59	-0.16 (-0.19, -0.12)	<0.0001
Unknown	2.78	-0.17 (-0.23, -0.11)	<0.0001
Female hormone usage (%)			
Yes	29.65	Reference	
No	67.25	0.02 (0.00, 0.04)	0.0483
Unknown	3.11	-0.01 (-0.06, 0.04)	0.6977
Long-term steroid usage (%)			
Yes	7.50	Reference	
No	91.96	0.02 (-0.01, 0.05)	0.1608
Unknown	0.54	-0.02 (-0.12, 0.09)	0.7304
Anti-osteoporosis treatment history (%)			
Yes	11.13	Reference	
No	9.62	0.08 (-0.01, 0.16)	0.0674
Unknown	79.25	0.23 (0.16, 0.29)	<0.0001

The proportion of menstruation status and female hormone usage were only calculated in the female group.

CI, confidence interval.

### Relationship between HDL-C and Intertrochanter BMD

**Figure 2:**
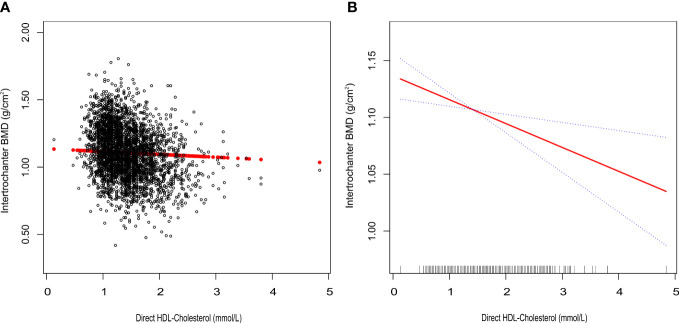
Association between HDL-C and Intertrochanter BMD. **(A)** each black point represents a sample. **(B)** the solid red line represents the smooth curve fit between variables. Blue bands represent the 95% confidence interval of the fit. All were adjusted for age, gender, race/ethnicity, body mass index, marital status, moderate activities, smoking status, diabetes status, cancer or malignancy, menstruation status, female hormone usage, long-term steroid usage, anti-osteoporosis treatment history, total calcium, phosphorus, total cholesterol, and total protein. The proportion of menstruation status and female hormone usage were only calculated in the female group.

We tracked a major negative connection between HDL-C and intertrochanter BMD (Model 1: β = -0.15, 95% CI: -0.17, -0.14. Model 2: β = -0.09, 95% CI: -0.10, -0.07. Model 3: β = -0.03, 95% CI: -0.04, -0.01; **Figure 2**; **Table 3**). In the fully adjusted model (Model 3), intertrochanter BMD decreased by 0.03 g/cm^2^ for every 1 mmol/L increase in HDL-C level. To further evaluate the association between HDL-C and intertrochanter BMD, we converted HDL-C values from a continuous variable to a classified variable (tertiles). The analysis revealed that, compared with the low HDL-C group (T1), the intertrochanter BMD in the high HDL-C group dropped by 0.03 g/cm^2^ for every increase of 1 mmol/L (*P* for trend = 0.019; **Table 3**).

**Table 3:** Relationship between HDL-C and Intertrochanter BMD.

Outcome	Model 1		Model 2		Model 3	
	*β* (95%CI)	*P* value	*β* (95%CI)	*P* value	*β* (95%CI)	*P* value
HDL-C (mmol/L)	-0.15 (-0.17, -0.14) <0.0001		-0.09 (-0.10, -0.07) <0.0001		-0.03 (-0.04, -0.01) 0.0002	
HDL-C categories						
T1(≤1.0 mmol/L)	Reference		Reference		Reference	
T2(1.0-2.0 mmol/L)	-0.09 (-0.10, -0.07) <0.0001		-0.04 (-0.06, -0.02) <0.0001		-0.00 (-0.02, 0.01)0.8098	
T3(>2.0 mmol/L)	-0.22 (-0.25, -0.20) <0.0001		-0.11 (-0.14, -0.09) <0.0001		-0.03 (-0.05, -0.01) 0.0107	
*P* for trend	<0.0001		<0.0001		0.019	

In sensitivity analysis, the HDL-C level was converted from a continuous variable to a categorical variable (tertiles).

Model 1: No covariates were adjusted.

Model 2: Adjusted for age, gender, and race/ethnicity.

Model 3: Adjusted for age, gender, race/ethnicity, body mass index, marital status, moderate activities, smoking status, diabetes status, cancer or malignancy, menstruation status, female hormone usage, long-term steroid usage, anti-osteoporosis treatment history, total calcium, phosphorus, total cholesterol, and total protein.

### Subgroup analysis

**Figure 3:**
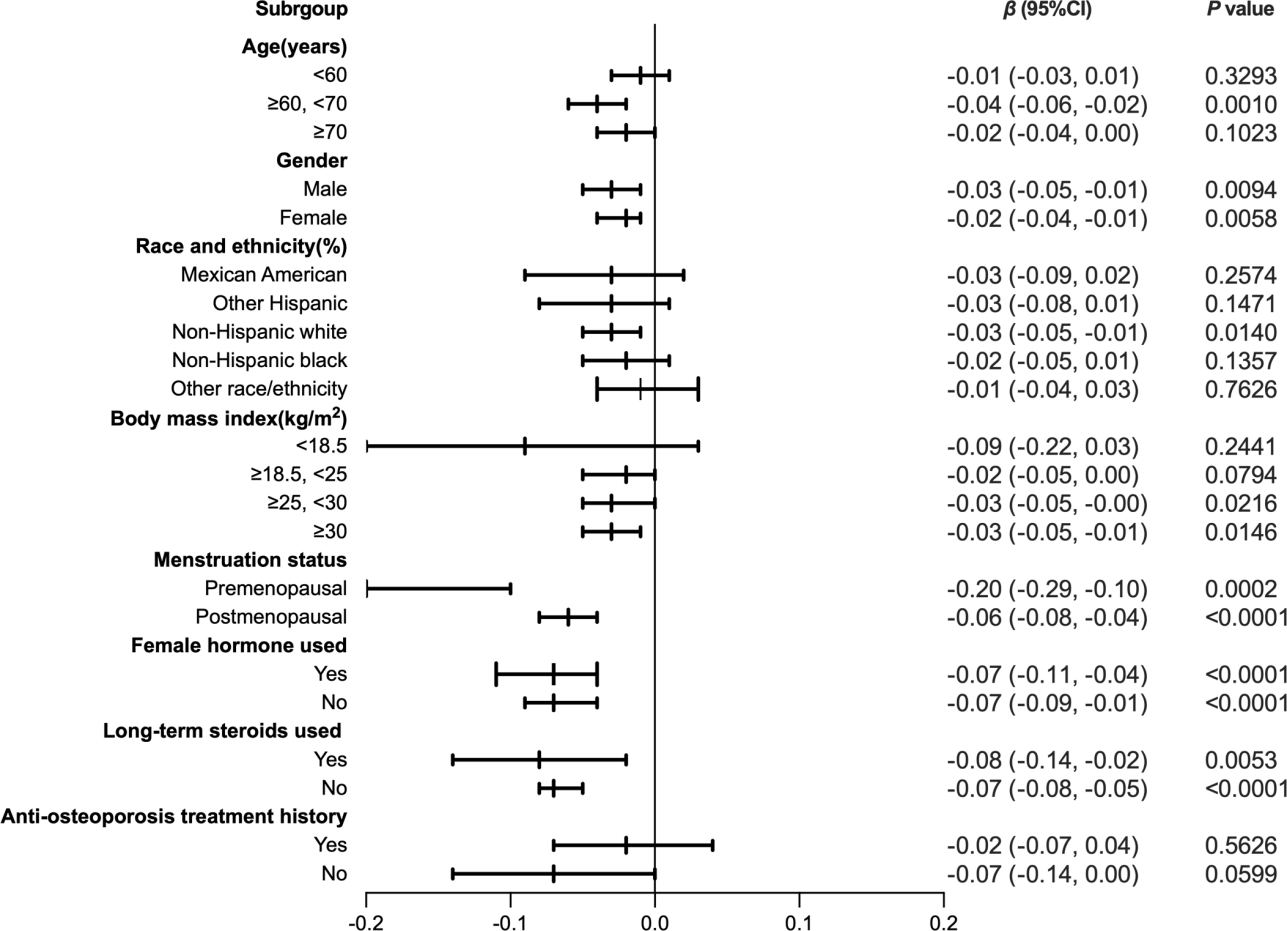
Forest plot analysis of subgroups for the association between HDL-C and Intratrochanter BMD.

**Figure 4:**
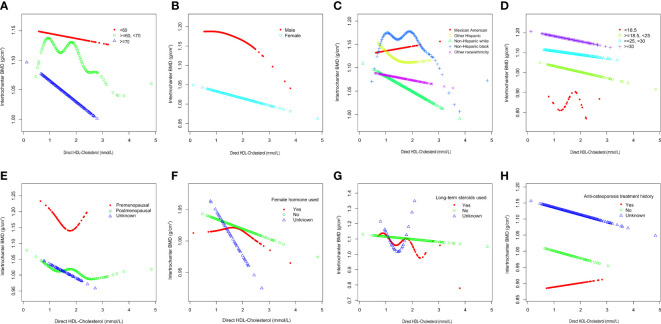
Subgroups analysis for the association between HDL-C and Intertrochanter BMD by **(A)** age, **(B)** gender, **(C)** race/ethnicity, **(D)** body mass index, **(E)** menstruation status, **(F)** female hormone usage, **(G)** long-term steroid usage, and **(H)** anti-osteoporosis treatment history. All were adjusted for age, gender, race/ethnicity, body mass index, marital status, moderate activities, smoking status, diabetes status, cancer or malignancy, menstruation status, female hormone usage, long-term steroid usage, anti-osteoporosis treatment history, total calcium, phosphorus, total cholesterol, and total protein, except the subgroup variable. The proportion of menstruation status and female hormone usage were only calculated in the female group.

To further assess the robustness of the association between HDL-C and intertrochanter BMD, subgroup analyses were performed by age, gender, race, BMI, menstruation status, female hormone usage, long-term steroid usage, and anti-osteoporosis treatment history (**Figure 3**). A negative association was discovered in subgroups of individuals aged 60-70 years (β = -0.04, 95% CI: -0.06, -0.02, *P* = 0.0010). Although the difference was not statistically significant among people over 70, the pattern of negative connection was pretty clear (**Figure 4A**). In subgroups stratified by gender, the inverse relationship was consistent across both male and female populations. Among subgroups split by race, the negative association was significant among non-Hispanic whites (β = -0.03, 95% CI: -0.05, -0.01, *P* = 0.0140). As a result, the same link was discovered in obese people in BMI subgroups (β = -0.03, 95% CI: -0.05, -0.01, *P* = 0.0146). Finally, when classified by menstruation status, female hormone usage, and long-term steroid usage, negative correlations were likewise detected for each subgroup (**Figure 3**). The association between HDL-C and Intertrochanter BMD was further confirmed by smooth curve fitting (**Figure 4**). Furthermore, we also evaluated the threshold effect of the non-linear relation (**Table 4**). In males and hormone users, the connection between HDL-C and intertrochanter BMD was an inverted U-shaped curve, with inflection points of 1.01 mmol/L and 1.71 mmol/L (**Figures 4B, F**). When HDL-C levels were < 1.01 mmol/L, the connection was not meaningful in men. Once the level of HDL-C surpassed the inaction point, it revealed a serious negative connection (β = -0.04, 95% CI: -0.07, -0.02, *P* = 0.0008). In female hormone users, the link was not substantial if HDL-C levels were lower than 1.71 mmol/L. And once the degree of HDL-C crossed the inflaction point, there existed a major negative connection (β = -0.12, 95% CI: -0.17, -0.07, *P* < 0.0001). Instead, it revealed a U-shaped curve in other Hispanic and premenopausal individuals, with the inflection point at 0.96 mmol/L and 1.89 mmol/L (**Figures 4C, E**). Other Hispanic and premenopausal individuals showed a negative correlation when HDL-C levels were below the inflection point, and only premenopausal individuals who were beyond the inflection point of 1.89 mmol/L indicated a positive association.

**Table 4:** Threshold effect analysis of high-density lipoprotein cholesterol on Intertrochanter BMD in different groups.

Outcome	Male		Other Hispanic		Premenopausal		Female hormone	
	β (95%CI)	*P* value	β (95%CI)	*P* value	β (95%CI)	*P* value	β (95%CI)	*P* value
Fitting by the standard linear model	-0.03 (-0.05, -0.01) 0.0094		-0.03 (-0.08, 0.01) 0.1471		-0.20 (-0.29, -0.10) 0.0002		-0.07 (-0.11, -0.04) <0.0001	
Fitting by the two-piecewise linear model								
Infection point	1.01		0.96		1.89		1.71	
HDL-C < Infection point	0.09 (-0.02, 0.20)0.1014		-0.53 (-0.89, -0.17) 0.0039		-0.27 (-0.41, -0.13)0.0004		0.05 (-0.01, 0.11)0.0848	
HDL-C > Infection point	-0.04 (-0.07, -0.02) 0.0008		-0.01 (-0.05, 0.04) 0.7448		0.57 (0.12, 1.02) 0.0162		-0.12 (-0.17, -0.07)<0.0001	
Log likelihood ratio	0.028		0.005		<0.0001		<0.0001	

## Discussion

In this cross-sectional study of 3,345 participants, we found a negative association between HDL-C and intertrochanter BMD. Subgroup analysis showed that this relationship was significant at ages 60-70, as well as among non-Hispanic whites and obese people, but was not altered by gender, menstruation status, female hormone usage, or long-term steroid usage history.

Dyslipidemia is a modifiable risk factor for the development and progression of cardiovascular disease and is characterized by raised low-density lipoprotein cholesterol (LDL-C) and triglycerides and decreased high-density lipoprotein cholesterol (HDL-C) (20). Recent trials have found that increasing HDL-C levels does not improve cardiovascular outcomes and suggest that high HDL-C levels may not be a protective factor for CVD (4, 5). These findings imply whether high HDL-C levels are associated with other specific populations or diseases.

The study on bone mineral density has become a hot issue (17, 21, 22), and most physicians or academics are examining the possible association between bone mineral density and other markers, although the results are conflicting. Maghbooli, Makovey, et al. found that HDL-C was inversely related to hip, femoral neck, and lumbar BMD in postmenopausal women (8, 9). According to Jiang et al., a low level of lumbar BMD and a high level of HDL-C were associated (10). Only postmenopausal women and the femoral neck and lumbar regions showed a negative correlation between HDL-C and BMD, according to Zhang et al (11). HDL-C was shown to be inversely linked with spine BMD in premenopausal women but not in postmenopausal women, according to Kim et al (18). Li et al. found that in the postmenopausal population, people with high levels of HDL-C tended to have lower femoral neck BMD or total hip BMD but not lumbar BMD (17). Go et al. found a positive correlation between HDL-C and femoral neck BMD (12). Zolfaroli et al. also found that HDL-C was positively correlated with femoral neck and lumbar BMD in postmenopausal women (13). Jeong et al. found a positive correlation between HDL-C and lunbar BMD in postmenopausal women and found that the relationship between HDL-C and BMD varied depending on BMD location (14). However, Cui et al.’s results do not support this view (15).

We observed that the results were inconsistent from study to study and that bone density was inconsistent across bone regions. These variations may be caused by demographic bias, the type of subgroup analysis used, changes in inclusion or exclusion criteria, or skeletal features at different sites. For instance, beyond age 60, the rate of bone loss in the lumbar area tends to slow or stabilize, and measures may be impacted by aortic sclerosis and degeneration. Conversely, bone deterioration in the hip and femur gets worse with age (7, 9).

In this analysis, we discovered that this negative connection changed by age, race, and BMI. Such individuals, ages 60–70, who are non-Hispanic whites and obese adults, should be alarmed. Conversely, the absence of distinction between the genders may be due to the selection of adults over the age of 50 and not encompassing all groups, which may have a selective bias. In obese individuals with a BMI > 30 kg/m^2^, such a major negative correlation may be attributed to the competing genesis of bone and fat cells from the same stem cell ancestor and the accumulation of excess fat leading to bone loss (23). Factors such as study sample size, participant diversity (male/female, ethnicity, vitamin D deficiency), and covariation-adjusted heterogeneity may have contributed to paradoxical results (13, 24, 25).

The intertrochanteric fracture was due primarily to the weakened impact resistance of the loose intertrochanteric area. When the bone density of such an intertrochanteric buffer zone deteriorated, the impact resistance decreased and the intertrochanteric fracture occurred. Research indicates that, after correcting for age, the death rates in patients with an intertrochanteric fracture after the accident were higher than those of the femoral neck fracture group at 1 year and 5 years (26, 27). Lower BMD in patients with an intertrochanteric fracture may be due to bone structure and an elevated PTH level (28).

Our study offers various advantages. First, this analysis relies on data from NHANES, a national, population-based sample obtained using standard protocols from reliable sources. Due to the large sample size, we can also conduct subgroup analysis split by age, gender, race, and BMI, menstruation status, female hormone usage, long-term steroid usage, and anti-osteoporosis treatment history to locate the essential group. In addition, we controlled for confounding factors to ensure that our results were credible. Finally, we performed threshold effect analysis to provide a more precise reference for clinical diagnosis and therapy.

The selection of covariables was based mainly on previous studies. Of course, the limitations of this study cannot be overlooked. First, the study was a cross-sectional design, and we were unable to obtain a causal relationship between HDL-C and intertrochanter BMD, which may be inspired by further basic research. Second, although we accounted for certain relevant confounders, we could not totally capture the influence of other possible confounding factors that may have had some impact on the results. Third, some indicators were too few to be included in the study, such as calcium supplements and lipid-lowering medications. Finally, our participants were over the age of 50, so our findings may not be generalizable to other populations.

## Conclusion

HDL-C is not inherently better. In this cross-sectional investigation, results demonstrated that HDL-C was negatively associated with intertrochanter BMD in adults over 50 years of age, non-Hispanic whites, and the obesity. Moreover, the underlying mechanism of such a correlation needs further exploration.

## Data availability statement

The datasets presented in this study can be found in online repositories. The names of the repository/repositories and accession number(s) can be found below: https://wwwn.cdc.gov/nchs/nhanes/continuousnhanes/default.aspx?cycle=2017-2020.

## Ethics statement

The studies involving human participants were reviewed and approved by NCHS Ethics Review Committee. The patients/participants provided their written informed consent to participate in this study.

## Author contributions

PY, and CL contributed to the conception and design of the study; PY contributed to manuscript writing; XL and DL contributed to data collection and management; ZT, and XN contributed to the statistical analysis; HW, YH and CL contributed to manuscript revision and data review. All authors contributed to the article and approved the submitted version.
